# Correction: The effect of *M*. *tuberculosis* lineage on clinical phenotype

**DOI:** 10.1371/journal.pgph.0003674

**Published:** 2024-08-23

**Authors:** Duc Hong Du, Ronald B. Geskus, Yanlin Zhao, Luigi Ruffo Codecasa, Daniela Maria Cirillo, Reinout van Crevel, Dyshelly Nurkartika Pascapurnama, Lidya Chaidir, Stefan Niemann, Roland Diel, Shaheed Vally Omar, Louis Grandjean, Sakib Rokadiya, Arturo Torres Ortitz, Nguyễn Hữu Lân, Đặng Thị Minh Hà, E. Grace Smith, Esther Robinson, Martin Dedicoat, Le Thanh Hoang Nhat, Guy E. Thwaites, Le Hong Van, Nguyen Thuy Thuong Thuong, Timothy M. Walker

Figs 1, 2, 4 and S4 Fig are incorrect. Please see the correct Figs 1, 2, 4 and S4 Fig here.

**Fig 1:**
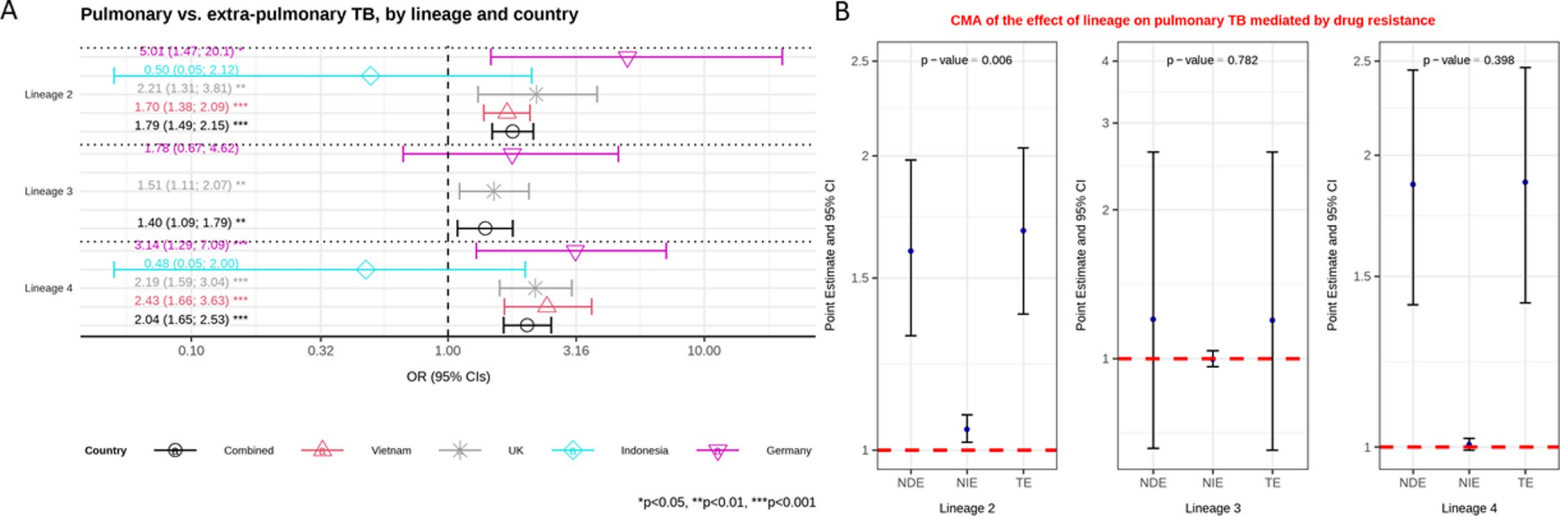
**A**: Multivariable logistic regression model on the association between lineage and pulmonary versus extra-pulmonary tuberculosis (TB) controlling for age and immigration. Estimated odd ratios (ORs) and bars representing 95% confidence intervals (CIs) are shown on the x-axis for lineage 2, 3 and 4, compared to lineage 1 as reference, for each country as well as for all these countries combined. P-values denote evidence of the associations of lineage and pulmonary TB. **B**: Causal mediation analysis (CMA) on the effect of lineage on pulmonary TB, mediated by drug resistance. Estimated odds ratio (ORs) and bars representing 95% confidence intervals (CIs) are shown on the y-axis for each decomposition effect including NDE: natural direct effect odds ratio; NIE: natural indirect effect odds ratio; and TE: total effect odds ratio for lineage 2, lineage 3, and lineage 4, compared to lineage 1 as reference. All multivariable models adjusted for country, immigration, and age are shown. P-values denote evidence of natural indirect effect of lineage on pulmonary TB mediated through drug resistance. The red horizontal lines indicate the thresholds of the results (ORs) of interest.

**Fig 2:**
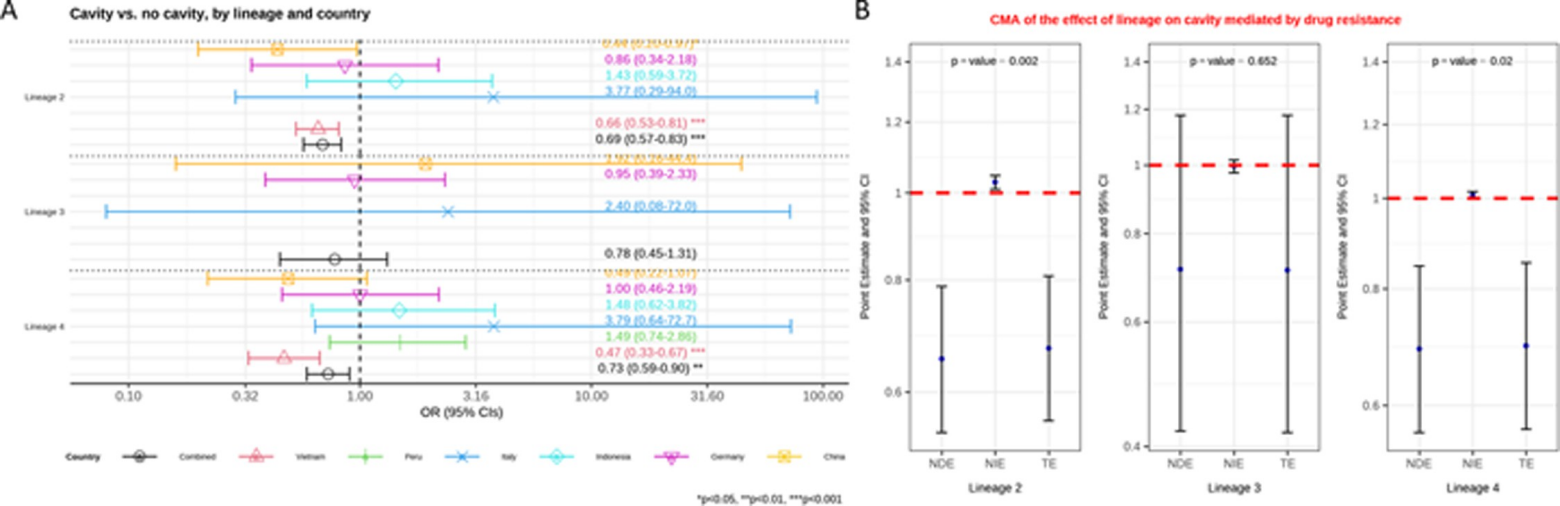
**A**: Multivariable logistic regression model on the association between lineage and the presence of cavity versus no cavity among patients with pulmonary TB, controlling for age and immigration. Estimated odd ratios (ORs) and bars representing 95% confidence intervals (CIs) are shown on the x-axis for lineage 2, 3 and 4, compared to lineage 1 as reference, for each country as well as for all these countries combined. P-values denote evidence of the associations of lineage and cavity. **B**: Causal mediation analysis (CMA) on the effect of lineage on cavity, mediated by drug resistance. Estimated odds ratio (ORs) and bars representing 95% confidence intervals (CIs) are shown on the y-axis for each of the decomposition effect including NDE: natural direct effect odds ratio; NIE: natural indirect effect odds ratio; and TE: total effect odds ratio of lineage 2, lineage 3, and lineage 4, compared to lineage 1 as reference. All multivariable models adjusted for country, immigration, and age are shown. P-values denote evidence of natural indirect effect of lineage on cavity mediated through drug resistance. The red horizontal lines indicate the thresholds of the results (ORs) of interest.

**Fig 4:**
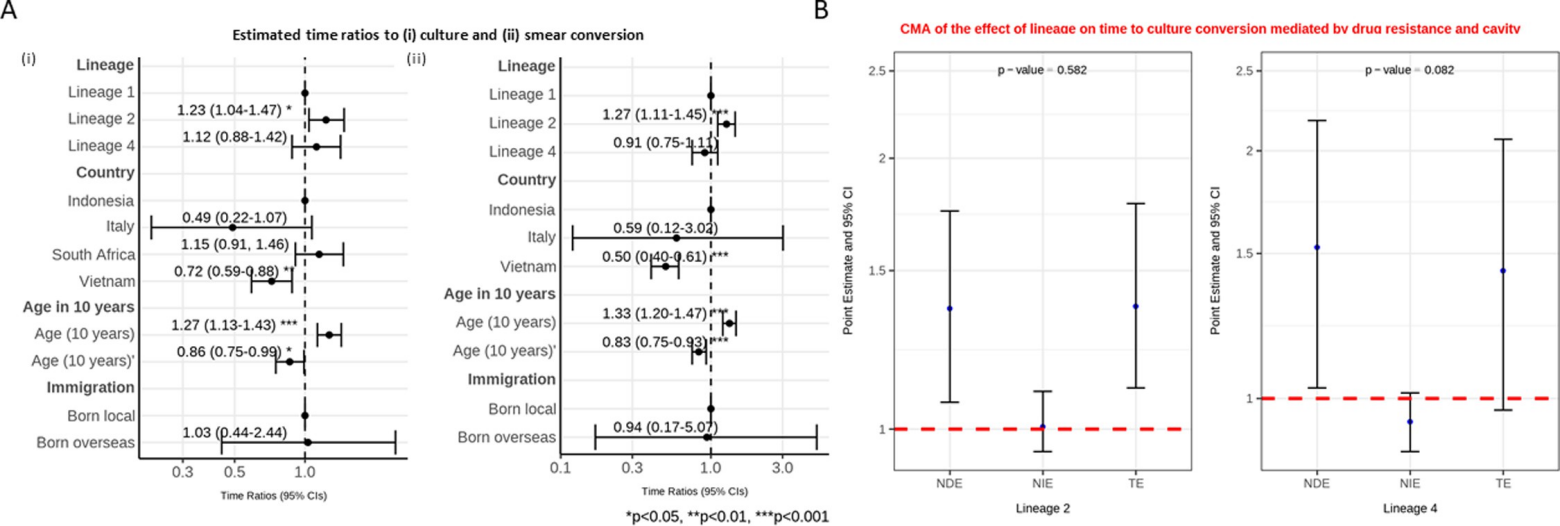
**A**: Interval censored regression using accelerated failure time models on the association between lineage and time to culture (i) and smear (ii) conversion controlling for age, country and immigration status. Estimated time ratios and bars representing 95% confidence intervals (CIs) are shown on the x-axis. Data from Indonesia, Italy and South Africa all had interval censored data whereas the data from Vietnam were binary (< = 60 days or >60 days). The Vietnamese data were therefore converted to interval data (“0 to 60” if < = 60; and “61 to ∞” if >60). P-values denote evidence of the associations of lineage and time to culture or smear conversion. **B**: Causal mediation analysis (CMA) on the effect of lineage on time to culture conversion mediated by drug resistance and cavity. Estimated time ratios and bars representing 95% confidence intervals (CIs) are shown on the y-axis for each of the decomposition effect including NDE: natural direct effect odds ratio; NIE: natural indirect effect odds ratio; and TE: total effect odds ratio of lineage 2 and lineage 4, compared to lineage 1 as reference. All multivariable models adjusted for country, immigration, and age are shown. P-values denote evidence of natural indirect effect of lineage on time to culture conversion mediated through drug resistance and cavity. The red horizontal lines indicate the thread holds of the results (ORs) of interest.

## Supporting information

S4 Fig Causal mediation analysis (CMA) on the effect of lineage on time to smear conversion mediated by drug resistance and cavity. Estimated time ratios and bars representing 95% confidence intervals (CIs) are shown on the y-axis for each of the decomposition effect including NDE: natural direct effect odds ratio; NIE: natural indirect effect odds ratio; and TE: total effect odds ratio of lineage 2 and lineage 4, compared to lineage 1 as reference. All multivariable models adjusted for country, immigration, and age are shown. P-values denote evidence of natural indirect effect of lineage on time to smear conversion mediated through drug resistance and cavity. The red horizontal lines indicate the thread holds of the results (ORs) of interest.
(TIF)
