# Supplementary figures and images for: The effect of M. tuberculosis lineage on clinical phenotype
Source: PLOS Glob Public Health. 2023 Dec 20;3(12):e0001788. doi: 10.1371/journal.pgph.0001788 (PMC10732390; doi:10.1371/journal.pgph.0001788)

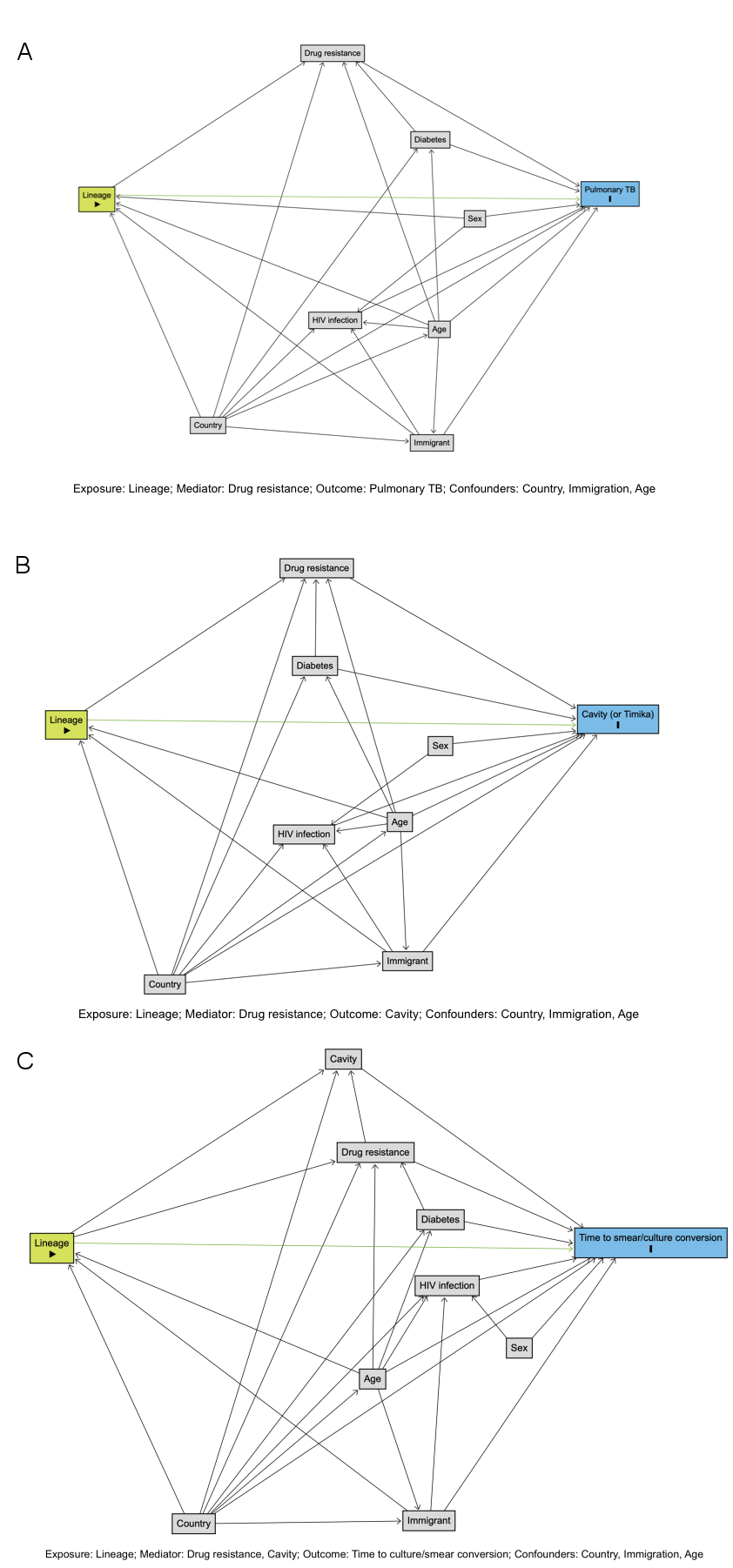

Supplement: S1 Fig — Arrows indicate the direction of the effect. Exposure, Mediator, Outcome and Confounders listed below each graph. (TIF) [file pgph.0001788.s001.tif]

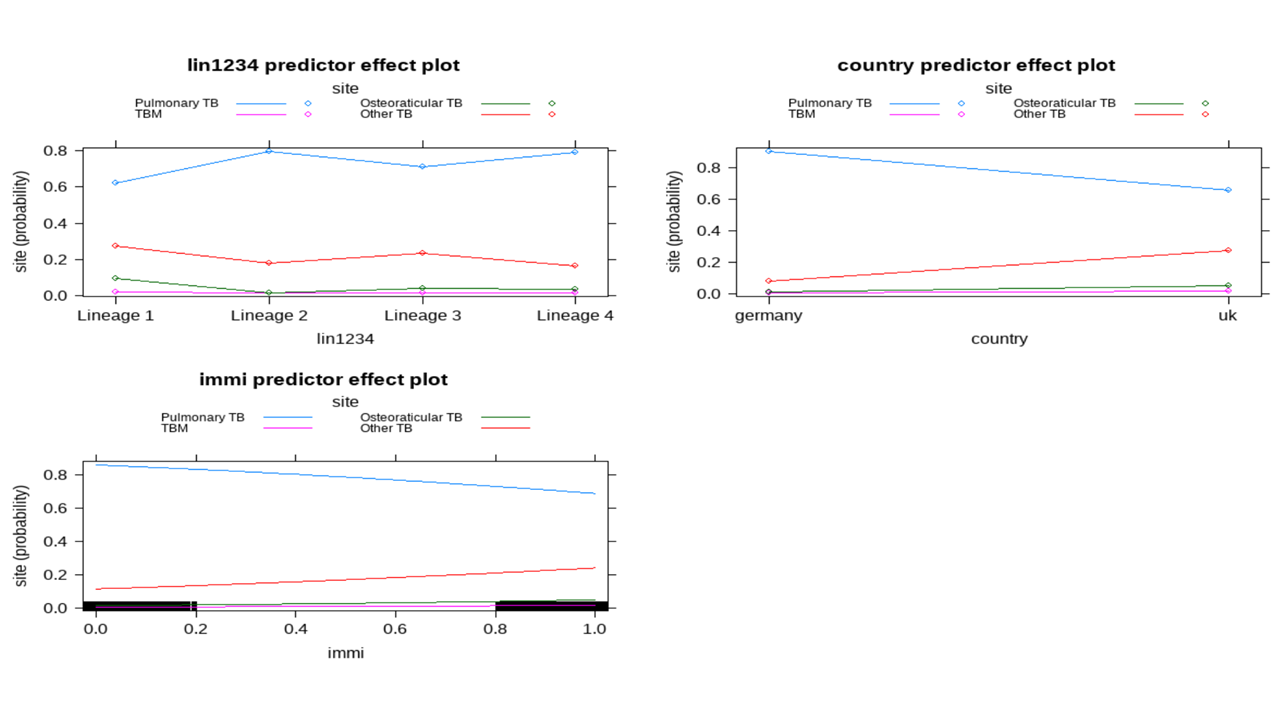

Supplement: S2 Fig — (TIF) [file pgph.0001788.s002.tif]

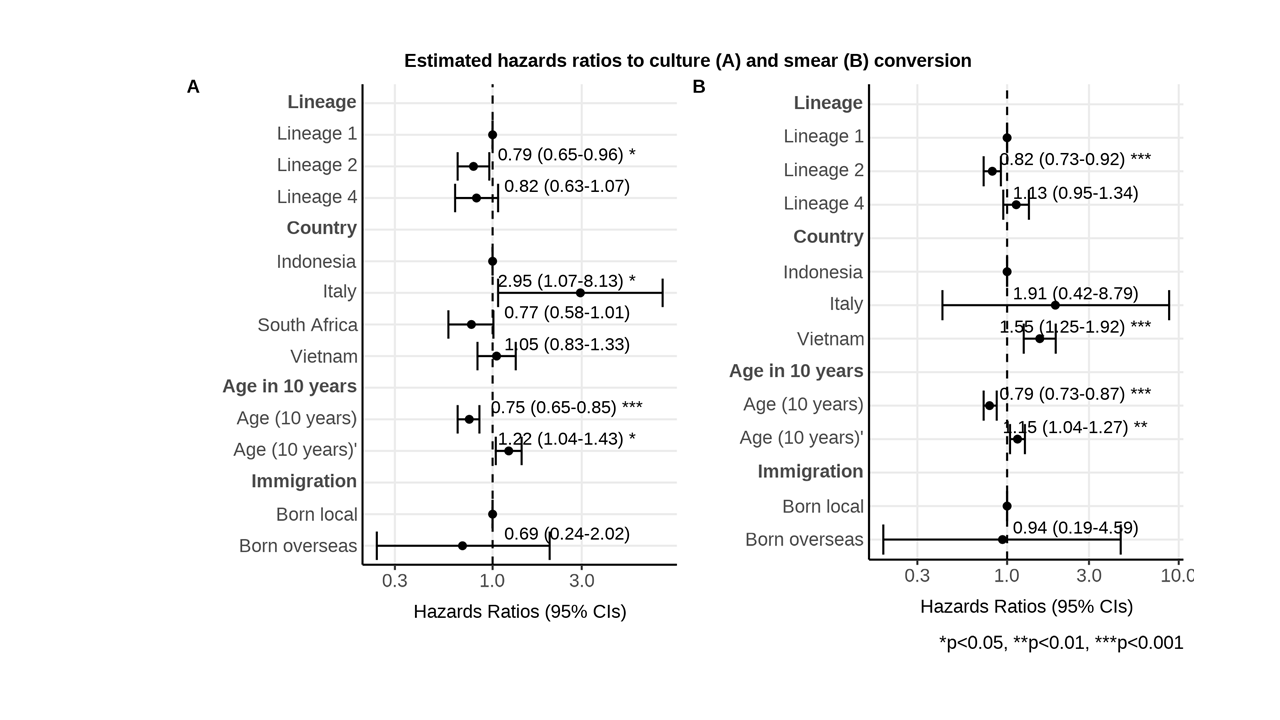

Supplement: S3 Fig — Estimated hazards ratios and bars representing 95% confidence intervals (CIs) are shown on the x-axis. Data from Indonesia, Italy and South Africa all had interval censored data whereas the data from Vietnam were binary (< = 60 days or >60 days). The Vietnamese data were therefore converted to interval data (“0 to 60” if < = 60; and “61 to ∞” if >60). P-values denote evidence of the associations of lineage and time to culture or smear conversion. (TIF) [file pgph.0001788.s003.tif]

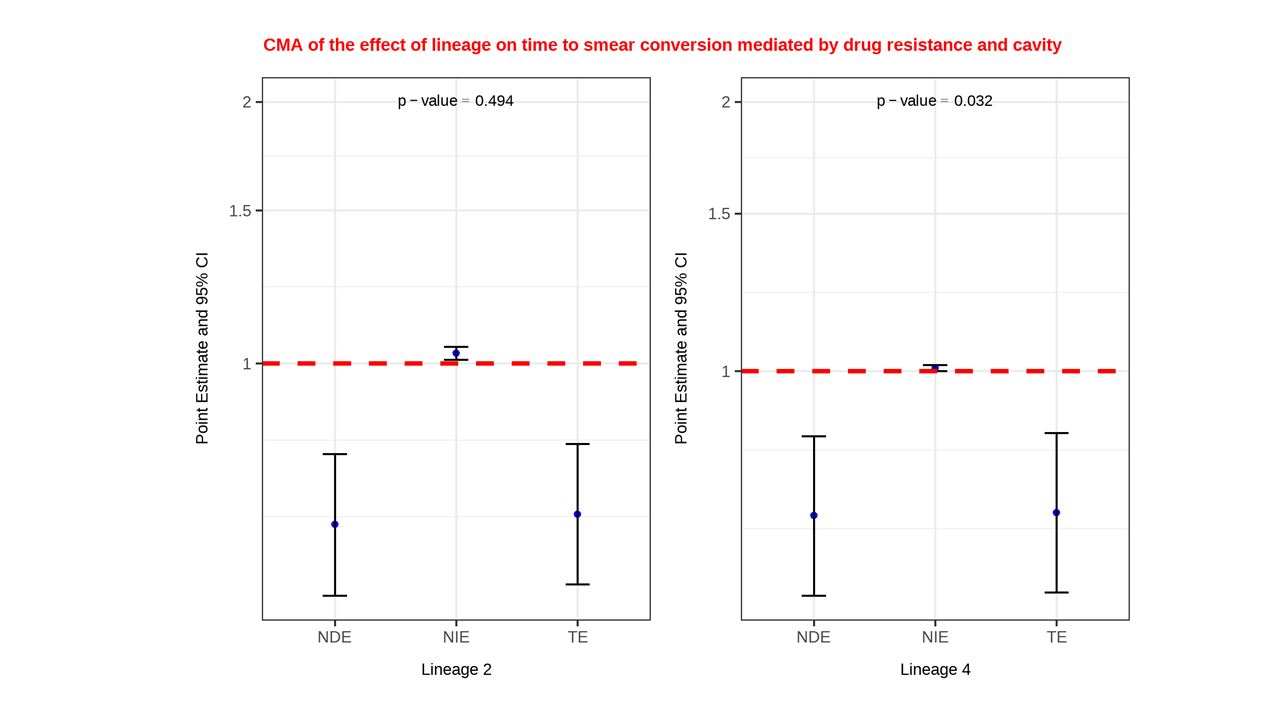

Supplement: S4 Fig — Estimated time ratios and bars representing 95% confidence intervals (CIs) are shown on the y-axis for each of the decomposition effect including NDE: natural direct effect odds ratio; NIE: natural indirect effect odds ratio; and TE: total effect odds ratio of lineage 2 and lineage 4, compared to lineage 1 as reference. All multivariable models adjusted for country, immigration, and age are shown. P-values denote evidence of natural indirect effect of lineage on time to smear conversion mediated through drug resistance and cavity. The red horizontal lines indicate the thread holds of the results (ORs) of interest. (TIF) [file pgph.0001788.s004.tif]
