# Supplementary material for: The effect of M. tuberculosis lineage on clinical phenotype
Source: PLOS Glob Public Health. 2023 Dec 20;3(12):e0001788. doi: 10.1371/journal.pgph.0001788 (PMC10732390; doi:10.1371/journal.pgph.0001788)
Supplement: Attachment — Submitted filename: Response To Reviewers.docx [file pgph.0001788.s024.docx]

Dear Editor,

Many thanks for the comments. Please find below our responses.

Best wishes,

Tim Walker

PGPH-D-23-00405
The effect of M. tuberculosis lineage on clinical phenotype: a retrospective observational study
PLOS Global Public Health

Dear Dr. Walker,

Thank you for submitting your manuscript to PLOS Global Public Health. After careful consideration, we feel that it has merit but does not fully meet PLOS Global Public Health’s publication criteria as it currently stands. Therefore, we invite you to submit a revised version of the manuscript that addresses the points raised during the review process.

After several rounds securing reviewers for your manuscript, we finally completed this process. My apologies on this delay.

Before your manuscript can be formally accepted you will need to complete some formatting changes, summarized here:

Introduction:

81. The objective of the study is well described, as well as the analytical framework necessary for the data and variables of interest.

Thank you. We are not sure what formatting changes are being requested here. Could you please clarify if any changes are indeed being requested?

Methods:

109. Reference is made to the use of the Timika Score for cavitations in chest X-rays. I recommend including an analysis of the disadvantages of this score and citing it. For example, in reference 14 punctuation is described but its disadvantages are not considered. Might consider: Chakraborthy A, Shivananjaiah AJ, Ramaswamy S, Chikkavenkatappa N. Chest X-ray score (Timika score): a useful adjunct in predicting treatment outcome in tuberculosis. Adv Respir Med. 2018;86(5):205-210. doi: 10.5603/ARM.2018.0032. PMID: 30378646.

We are again not entirely sure what is being requested here. Reading the above referenced paper, the Timika score is found to be quite useful. The only ‘disadvantage’ it identifies is that in another referenced manuscript the score doesn’t correlate with smear. Is that what we are being asked to add to our manuscript? It’s not immediately clear to us how that is relevant to our analysis. Please do clarify if we have misunderstood. Many thanks.

The statistical analyzes look adequate and consider avoiding bias in the interpretation of the results. Especially when considering the role of lineage 1 in the extrapulmonary manifestations of TB and the greater transmissibility associated with pulmonary TB in the other lineages.

Thank you.

Results:

204. As mentioned in the introduction, there are other TB lineages identified, however, for reasons also explained by the authors in this section, these lineages have not been included because they are very rare or poorly represented, however, for didactic reasons I recommend naming them and location.

We have added a description of the lineages and the country of origin of the remaining isolates (see line 207). 6 were M africanum and 1 was M bovis.

Discussion:

423. In this section the authors make some interesting conclusions, especially the possibility that lineages 2, 3 and 4 are associated with pulmonary manifestations and therefore with greater transmissibility. I would recommend placing some reference or including a sentence regarding this important fact, especially considering that in other Latin American countries where there is transmission and circulation of MDR-TB, such as Haiti and the Dominican Republic, this could open the possibility of future phylogenetic analysis. of TB and how TB control programs can focus their strategies.

This refers to the primary findings of our analysis. In the discussion we make reference to a number of corroborating studies, such as in lines 423-428: “Lineage 1 is ancestral to the other 3 lineages assessed in this study (25). It is most prevalent in south Asia and has been linked to both extra-pulmonary TB and to more severe disease phenotypes (9,26,27). It is plausible that the modern lineages (2, 3 and 4) are more adapted to manifesting as pulmonary TB, thereby favouring more transmission(10). Indeed, there is evidence from Vietnam and elsewhere that lineage 1 is being out-competed by the other three global lineages (28,29).” We have added one more sentence (and reference) here to highlight some implications for MDR TB which appears to be less common in L1.

In general, I congratulate the authors for this work, I hope they can do another work where the genomic characteristics of this lineage and its role in the expression of the disease can be analyzed in depth.

Guidelines for resubmitting your figure files are available below the reviewer comments at the end of this letter.

We look forward to receiving your revised manuscript.

Kind regards,

Marianne Clemence, Staff Editor, on behalf of,

Robert Paulino-Ramirez
Staff Editor
PLOS Global Public Health

Journal Requirements:

1. Please include a complete copy of PLOS’ questionnaire on inclusivity in global research in your revised manuscript. Our policy for research in this area aims to improve transparency in the reporting of research performed outside of researchers’ own country or community. The policy applies to researchers who have travelled to a different country to conduct research, research with Indigenous populations or their lands, and research on cultural artefacts. The questionnaire can also be requested at the journal’s discretion for any other submissions, even if these conditions are not met.  Please find more information on the policy and a link to download a blank copy of the questionnaire here: <https://journals.plos.org/globalpublichealth/s/best-practices-in-research-reporting>. Please upload a completed version of your questionnaire as Supporting Information when you resubmit your manuscript.

Completed and uploaded.

2. Please update your online Competing Interests statement. If you have no competing interests to declare, please state: “The authors have declared that no competing interests exist.”

“The authors have declared that no competing interests exist.”

3. Please provide separate figure files in .tif or .eps format only and ensure that all files are under our size limit of 10MB.

For more information about how to convert your figure files please see our guidelines: <https://journals.plos.org/globalpublichealth/s/figures>

Done.

4. Please ensure that the Title in your manuscript file and the Title provided in your online submission form are the same.

Yes, it is.

5. We have noticed that you have uploaded Supporting Information files, but you have not included a list of legends. Please add a full list of legends for your Supporting Information files after the references list.

The legends for all the supplementary figures are embedded in the manuscript following the online guidance. We have now provided legends for each of the supplementary tables as well.

6. We notice that your supplementary figures are uploaded with the file type 'Figure'. Please amend the file type to 'Supporting Information'. Please ensure that each Supporting Information file has a legend listed in the manuscript after the references list.

Done

7. Please review your reference list to ensure that it is complete and correct. If you have cited papers that have been retracted, please include the rationale for doing so in the manuscript text, or remove these references and replace them with relevant current references. Any changes to the reference list should be mentioned in the rebuttal letter that accompanies your revised manuscript. If you need to cite a retracted article, indicate the article’s retracted status in the References list and also include a citation and full reference for the retraction notice.

We have not, to the best of our knowledge, cited any retracted papers.

Additional Editor Comments (if provided):


[Note: HTML markup is below. Please do not edit.]

Reviewers' comments:

Reviewer's Responses to Questions

**Comments to the Author**

1. Does this manuscript meet PLOS Global Public Health’s [publication criteria](https://journals.plos.org/globalpublichealth/s/criteria-for-publication)? Is the manuscript technically sound, and do the data support the conclusions? The manuscript must describe methodologically and ethically rigorous research with conclusions that are appropriately drawn based on the data presented.

Reviewer #1: Yes

Reviewer #2: Yes

2. Has the statistical analysis been performed appropriately and rigorously?

Reviewer #1: Yes

Reviewer #2: Yes

3. Have the authors made all data underlying the findings in their manuscript fully available (please refer to the Data Availability Statement at the start of the manuscript PDF file)?

The [PLOS Data policy](https://journals.plos.org/globalpublichealth/s/data-availability) requires authors to make all data underlying the findings described in their manuscript fully available without restriction, with rare exception. The data should be provided as part of the manuscript or its supporting information, or deposited to a public repository. For example, in addition to summary statistics, the data points behind means, medians and variance measures should be available. If there are restrictions on publicly sharing data—e.g. participant privacy or use of data from a third party—those must be specified.

Reviewer #1: Yes

Reviewer #2: Yes

4. Is the manuscript presented in an intelligible fashion and written in standard English?

PLOS Global Public Health does not copyedit accepted manuscripts, so the language in submitted articles must be clear, correct, and unambiguous. Any typographical or grammatical errors should be corrected at revision, so please note any specific errors here.

Reviewer #1: Yes

Reviewer #2: Yes

5. Review Comments to the Author

Please use the space provided to explain your answers to the questions above. You may also include additional comments for the author, including concerns about dual publication, research ethics, or publication ethics. (Please upload your review as an attachment if it exceeds 20,000 characters)

Reviewer #1: Interesting paper reinforcing the evidence on lineage effects impacting clinical phenotypes. I appreciate the efforts made to explain the rationale behind the pooling of other existing study datasets, including previously published studies from the same authors (e.g. ref 23) that did not investigate this particular manuscript's hypotheses.

Although using very diverse and not necessarily comparable datasets at first, the authors appropriately tailored their analyses to extract relevant and statistically valid interpretations, and clearly delineated the limitations of their approach and conclusions.

I have no hesitation to recommend this manuscript for publication. I have a very minor detail to raise, not that it should impact the recommendation: the abstract refers to 8 lineages of MTB sensu stricto but I'm only aware of 6 lineages (1, 2, 3, 4, 7, 8), although the authors might be more in-the-know as to the latest lineages discovered in the recent year.

We thank the reviewer for the positive remarks. The reviewer is correct to say that lineages 5 and 6 (M. africanum 1 and 2) are not actually MTB sensu stricto. Indeed, a 9^th^ lineage (another M africanum) has recently been described (*Microb Genom*. 2021;7:1–14. doi: 10.1099/mgen.0.000477). To avoid confusion, we have changed ‘8’ to ‘6’ and added a caveat to remind the reader that this exclude M africanum.

Reviewer #2: Grateful for the opportunity to review the manuscript "The effect of M. tuberculosis lineage on clinical phenotype: a retrospective observational study" (manuscript number PGPH-D-23-00405 ).
The study was well designed, and the findings were adequately presented and discussed, including the authors doing very well to acknowledge and carefully describe the limitations of the study.
In general, studies on the association between M. tuberculosis strain and clinical phenotype are important and should be treated with caution. Congratulations to the authors for the excellent work!
I recommend publishing this manuscript in PLOS Global Public Health.

Thank you!
