# Supplementary material for: The effect of M. tuberculosis lineage on clinical phenotype
Source: PLOS Glob Public Health. 2023 Dec 20;3(12):e0001788. doi: 10.1371/journal.pgph.0001788 (PMC10732390; doi:10.1371/journal.pgph.0001788)
Supplement: Attachment — Submitted filename: Review of Walker et al.docx [file pgph.0001788.s025.docx]

Review of Walker *et al*, The effect of M. tuberculosis lineage on clinical phenotype

The authors conduct a retrospective observational analysis pooling international datasets to quantify the effect of *M. tuberculosis* lineage on clinical disease presentation. I congratulate the authors on this important piece of work and appreciate the causal inference approach they took. Pooling observational data from diverse settings and countries is challenging and the authors did well in voicing the limitations of this approach.

I have a few comments below to improve the manuscript:

1. The authors could comment on the advantages of using causal mediation analysis to quantify effects of mediators as opposed to multivariate models.
2. Second generation immigration, i.e. where country of birth is UK or Germany but likely frequent travel to the parents’ country of birth, could also be an important confounder.
3. Please use larger fonts in figures
4. Briefly describe on your imputation approach in the methods section
5. spelling error L288: that ‘it’ might behave similar
6. *‘TB osteomyelitis was significantly more likely than non-meningeal foci of extra-pulmonary TB (“other”) for lineage 1 as compared to lineages 2, 3 and 4 (p=0.032, p=0.01, and p=0.049 respectively*. This sentence is unclear since TB osteomyelitis is a non-meningeal focus?
7. *These intrinsic biases need to be recognised when constructing each of the presented models. However, none of the studies we pooled data from selected on the basis of lineage as such data could not be known until after the isolates had been analysed. As such each collection had the opportunity to inform on the distribution of lineages within it.*

The authors adequately described the limitations of pooling data from different studies for their purpose, i.e., by presenting effect estimates from country specific models. However, I think their last statement above is incorrect. The different selected studies, i.e. those studying drug resistant or meningeal TB, will be biased towards lineages if the authors assumption is that underlying lineages differ in resistance profiles and disease presentation.
