# Supplementary material for: The effect of M. tuberculosis lineage on clinical phenotype
Source: PLOS Glob Public Health. 2023 Dec 20;3(12):e0001788. doi: 10.1371/journal.pgph.0001788 (PMC10732390; doi:10.1371/journal.pgph.0001788)
Supplement: Attachment — Submitted filename: Response to Reviewers.docx [file pgph.0001788.s026.docx]

Review of Walker *et al*, The effect of M. tuberculosis lineage on clinical phenotype

The authors conduct a retrospective observational analysis pooling international datasets to quantify the effect of *M. tuberculosis* lineage on clinical disease presentation. I congratulate the authors on this important piece of work and appreciate the causal inference approach they took. Pooling observational data from diverse settings and countries is challenging and the authors did well in voicing the limitations of this approach.

Thank you.

I have a few comments below to improve the manuscript:

1. The authors could comment on the advantages of using causal mediation analysis to quantify effects of mediators as opposed to multivariate models.

We have elaborated in lines 152-156: “We conducted causal mediation analyses to investigate the key drivers of our selected outcomes. To this end we focussed on pathways of effects from lineage on outcome, quantifying the effect-size attributable to mediators, rather than just assessing the marginal effect of lineage by standard multivariate models alone.”

2. Second generation immigration, i.e. where country of birth is UK or Germany but likely frequent travel to the parents’ country of birth, could also be an important confounder.

This is true but we think it is less of a confounder than overseas birth as living in a high-burden country typically leads to more exposure than just visiting family there on occasion. However, we had no data on 2^nd^ generation immigration and could not assess this unfortunately. We have added this point to the limitations section in the discussion.

3. Please use larger fonts in figures

Done

4. Briefly describe on your imputation approach in the methods section

We have added a paragraph from line 160: “Direct counterfactual imputation estimation was conducted by fitting a model for the outcome conditional on exposure (A), mediator (M) and covariates (C) using the original dataset, E(Y|A,M,C). For each level of the mediator, we fitted a regression model for the distribution of that mediator, conditional on exposure and covariates, and simulated the counterfactuals of the mediator based on the active and control values for the exposure. We then imputed the counterfactuals of outcome for each observation conditional on exposure, mediator and covariates, given each level of the mediator and the predict function from the initial regression model. An average was taken to obtain the counterfactual estimation, and causal -, direct -, indirect -, and total-effects were calculated.”

5. spelling error L288: that ‘it’ might behave similar

Corrected.

6. *‘TB osteomyelitis was significantly more likely than non-meningeal foci of extra-pulmonary TB (“other”) for lineage 1 as compared to lineages 2, 3 and 4 (p=0.032, p=0.01, and p=0.049 respectively*. This sentence is unclear since TB osteomyelitis is a non-meningeal focus?

Sorry, this was confusingly phrased. The sentence before says that we looked as “TB meningitis, osteomyelitis or other forms of extra-pulmonary TB”. So we have changed the sentence in question to align better with this: “TB osteomyelitis was significantly more likely than other non-meningeal foci of extra-pulmonary TB for lineage 1 as compared to lineages 2, 3 and 4”.

7. *These intrinsic biases need to be recognised when constructing each of the presented models. However, none of the studies we pooled data from selected on the basis of lineage as such data could not be known until after the isolates had been analysed. As such each collection had the opportunity to inform on the distribution of lineages within it.*

The authors adequately described the limitations of pooling data from different studies for their purpose, i.e., by presenting effect estimates from country specific models. However, I think their last statement above is incorrect. The different selected studies, i.e. those studying drug resistant or meningeal TB, will be biased towards lineages if the authors assumption is that underlying lineages differ in resistance profiles and disease presentation.

We can see how this sentence might have led to confusion. The point we were trying to make in was that selection bias by lineage was improbable within any cohort as lineage wouldn’t have been know at the time of selection. Thus, any skew towards a particular lineage in that population would be genuinely reflective of lineage X being more likely to cause outcome Y. We have re-phrased this to clarify:

“As such, the distribution of lineages within each collection should reflect the lineages’ natural tendency to result in the outcomes in question, and not any extrinsic selection bias”

We hope that clarifies things better.
