# Supplementary figures and images for: Correction: The effect of M. tuberculosis lineage on clinical phenotype
Source: PLOS Glob Public Health. 2024 Aug 23;4(8):e0003674. doi: 10.1371/journal.pgph.0003674 (PMC11343449; doi:10.1371/journal.pgph.0003674)

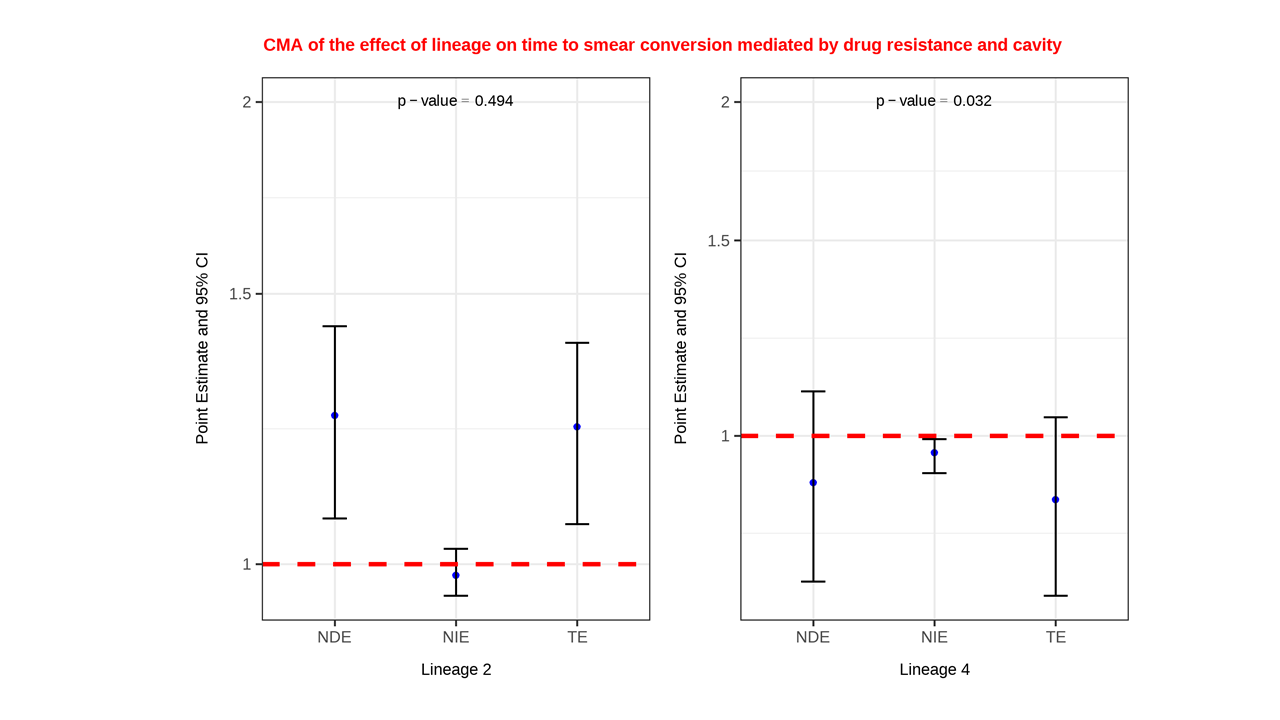

Supplement: S4 Fig — Estimated time ratios and bars representing 95% confidence intervals (CIs) are shown on the y-axis for each of the decomposition effect including NDE: natural direct effect odds ratio; NIE: natural indirect effect odds ratio; and TE: total effect odds ratio of lineage 2 and lineage 4, compared to lineage 1 as reference. All multivariable models adjusted for country, immigration, and age are shown. P-values denote evidence of natural indirect effect of lineage on time to smear conversion mediated through drug resistance and cavity. The red horizontal lines indicate the thread holds of the results (ORs) of interest. (TIF) [file pgph.0003674.s001.tif]
